# Alternative Pathways: The Use Of Field-Specific Micro-Credentials in Aging Services

**DOI:** 10.1093/geroni/igab046.2807

**Published:** 2021-12-17

**Authors:** M Aaron Guest, Leanne Clark-Shirley, Cynthia Hancock, Tina K Newsham, Katherine Alvarado, Kristina Amirchian Renee DuMont, Lauren Hackett, Kaylah Jenkins J R Patton, Caylee Weaver

**Affiliations:** 1 Arizona State University, Tempe, Arizona, United States; 2 American Society on Aging, American Society on Aging, California, United States; 3 University of North Carolina Charlotte, Charlotte, North Carolina, United States; 4 University of North Carolina Wilmington, University of North Carolina Wilmington, North Carolina, United States; 5 University of North Carolina Wilmington, Wilmington, North Carolina, United States

## Abstract

The rise of formal academic programs in gerontology at colleges and universities has been well documented over the last fifty years. Organizations such as AGHE and AGEC have been established to provide guidance, foster consistency, and advance formal gerontology education programs. Broadly, the purpose of these programs has been to develop a pipeline of trained gerontologists for the aging services workforce. What has been less documented is the rise of alternative pathways to gerontology and gerontological competence, including micro-credentialing. Micro-credentials are intended to provide quick-to-complete competency-based education around specific topics to demonstrate relevant skills to employers. To date, little is known about the prevalence of micro-credentialing in gerontology. Still, it may be that micro-credentials are sought in place of formal academic preparation due to their reduced cost, ease of completion, recognizability, and opportunity to quickly train employees in specific skills. To address this gap, we conducted a review of existing gerontological micro-credentialing opportunities. We identified a total of 51 micro-credentials with an explicit aging-focus and searched for associated competencies for these micro-credentials. In this poster, we describe findings on the emphases of micro-credentials, including dementia and care coordination, and review the programs' scope and nature of competencies. We argue that micro-credentialing can offer value for employees unable or unwilling to pursue formal academic training but should be differentiated from such training. Moving forward, it is critical to ensure alignment between gerontological micro-credentials and established gerontological competencies and standards and to differentiate micro-credentials from formal academic programs.

